# Interactions of Polyphenolic Compounds with Gelling Agents: Health-Promoting Properties and Application in Food Systems

**DOI:** 10.3390/gels12010030

**Published:** 2025-12-30

**Authors:** Natalia Żurek, Greta Adamczyk

**Affiliations:** Department of Food Technology and Human Nutrition, Faculty of Technology and Life Sciences, University of Rzeszow, 4 Zelwerowicza St., 35-601 Rzeszow, Poland; gadamczyk@ur.edu.pl

**Keywords:** polyphenols, biological activity, gelling agents, bioavailability, hydrocolloids, carbohydrates

## Abstract

Despite their numerous advantages, polyphenolic compounds are characterized by low bioavailability during gastrointestinal digestion and high sensitivity to technological processes during food preparation and storage. Therefore, numerous studies conducted over the past decade have identified overcoming these challenges as the undisputed goal of fully utilizing their functional properties. One solution to these challenges is the use of gelling agents. Their use as carriers for polyphenolic compounds has improved their stability during digestion and subsequent technological applications. This study analyzed the latest available scientific reports to determine the effect of combining polyphenolic compounds with gelling agents on the bioavailability, biological activity, and subsequent technological applications of the resulting ingredients.

## 1. Introduction

Polyphenolic compounds are a group of secondary metabolites generated by plants in response to environmental stress, performing protective functions against biotic and abiotic factors [1]. Chemically, they are composed of at least one aromatic ring and two or more hydroxyl groups. Five classes of these compounds can be distinguished: phenolic acids, flavonoids (anthocyanins, flavanols, flavonols, flavanones, isoflavones), stilbenes, lignans, and tannins. Each of these classes differs significantly in terms of physicochemical and functional properties. Polyphenolic compounds are present in various morphological parts of plant materials, including flowers, fruits, seeds, leaves, stems, bark, and roots [2,3,4]. In the human diet, their main sources are fruits, vegetables, cereals, nuts, plant drinks and spices [5]. For centuries, polyphenols have also been an important focus of research in the context of health-promoting properties, being the subject of interest of the medical, cosmetic, nutraceutical and food industries [6]. In vitro and in vivo studies have shown a significant correlation between the consumption of polyphenols and a reduced risk of developing diseases such as diabetes, obesity, cardiovascular diseases, cancer, diseases caused by chronic inflammation, and bacterial infections [7,8]. However, despite these numerous advantages, polyphenols (mainly flavonoids) are among the compounds with low stability and bioavailability during digestion in the gastrointestinal tract, as well as high sensitivity to technological processes during food preparation and storage. Therefore, in numerous studies over the last decade, overcoming these challenges has become an undoubted task in order to fully utilize their health-promoting properties [9,10].

**Figure 1:**
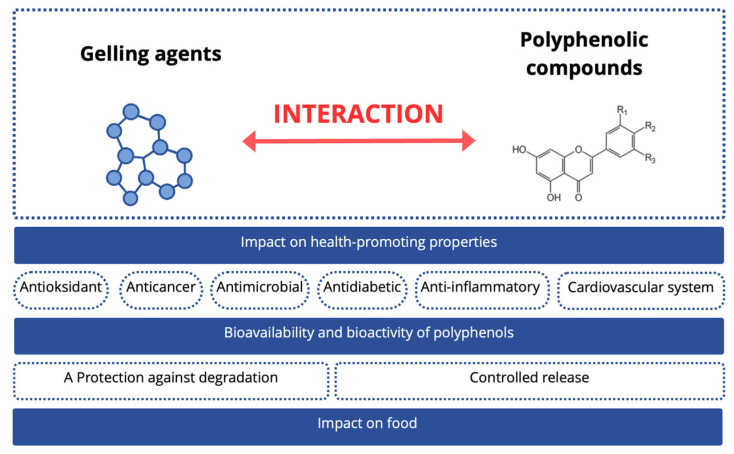
Interactions between polyphenolic compounds and gelling agents.

Gelling agents are substances that have the ability to form gels, which are three-dimensional network structures capable of retaining fluids. A characteristic feature of gels is that they possess the properties of both solids (texture, shape) and liquids (moisture, elasticity) [11]. The properties of gelling materials are a result of the interaction between these two components. Based on their chemical nature, gelling agents can be classified as polysaccharides (pectins, alginates, agar, vegetable gums, carrageenans, hyaluronic acid), proteins (gelatin, caseinates), starches (potato starch, corn starch, tapioca starch, modified starches), and synthetic and semi-synthetic agents (carboxymethylcellulose (CMC), methylcellulose (MC), hydroxypropylmethylcellulose (HPMC), sodium polyacrylate) [12]. These substances differ in their gelation mechanisms. Gelation can occur physically, by changing the solution’s pH, changing temperature, using salt ions, or through enzyme activity. Chemical methods of gel formation can also be distinguished, including induction with metal ions and organic compounds, as well as the latest methods, such as high pressure, ultrasound, and microwaves [13]. Gels obtained in this way play a key role in many applications in the food, pharmaceutical and cosmetics industries; packaging design; tissue engineering; and wound dressings (Figure 1) [14].

In recent years, gelling agents combined with polyphenolic compounds have been extensively studied for their impact on the bioavailability and bioactive properties of absorbed polyphenols, as well as on the mechanisms of formation and stability of gel structures [15,16]. Depending on the gel type, polyphenolic compounds can be soluble in the gel matrix, adsorbed on its surface, encapsulated in the core, or immobilized in a polymer network. Such strategies can potentially modify the stability, bioavailability, and biological activity of polyphenols, as well as influence the rheological and structural properties of gel matrices, determining their behavior during processing, storage, and digestion. Ultimately, these interactions influence the potential use of the resulting formulations as food products. However, despite numerous studies, a recent report combining these achievements is still lacking.

Therefore, the aim of this work was to thoroughly analyze the latest research reports on the impact of systems composed of polyphenolic compounds and gelling agents on their functional and health-promoting properties, as well as their bioavailability during gastrointestinal digestion. Furthermore, the nature and mechanisms of interactions between polyphenols and gel matrices are discussed; their impact on the rheological, structural, biological, and technological properties of gels is assessed; and the latest breakthroughs in their application in food production are presented. We believe that this report, developed in accordance with this goal, will represent an important addition to our current knowledge on polyphenol–gelling agent systems and be important for both the scientific and technological communities.

## 2. Results and Discussion

### 2.1. Mechanisms of Interaction Between Polyphenols and Gelling Agents

Gelling agents such as polysaccharides, proteins and synthetic polymers interact with polyphenolic compounds mainly through hydrogen bonds, electrostatic, hydrophobic and π-π interactions [17]. These interactions directly impact the physicochemical, nutritional, functional, sensory, and safety properties of the resulting formulations. Hydrogen bonding occurs primarily between the hydroxyl groups of polyphenols and the carboxyl, hydroxyl, or amine groups of gelling agents [18]. Hydrophobic interactions occur between the aromatic rings of polyphenols and nonpolar fragments of protein chains or hydrophobic regions of polysaccharides [19]. Electrostatic interactions, in turn, occur between the ionized phenolic groups of polyphenols and charged functional groups in gelling materials, such as positively charged amino groups in proteins or negatively charged carboxyl groups in polysaccharides. In contrast, π-π arrangements are generated between the aromatic rings of polyphenols and aromatic amino acid residues in proteins (e.g., phenylalanine, tryptophan, tyrosine) [20]. These interactions typically interact to form reversible or irreversible complexes. They also determine their chemical and physical behavior, causing changes in food color, flavor, and turbidity, ultimately impacting their potential applications. They also modulate the bioavailability of polyphenols and their storage and thermal stability. Furthermore, the structure of polysaccharides, proteins, and polyphenols, their concentration, temperature, pH, processing time, and their ionic strength can influence the degree of binding and stability of these interactions [21]. Identification and evaluation of the strength, adsorption kinetics, and structural changes in interactions between polyphenols and gelling agents can be performed using Fourier transform infrared spectroscopy (FT-IR), size exclusion chromatography, X-ray diffraction, ultraviolet-visible spectrophotometry, atomic force microscopy, transmission electron microscopy (TEM), scanning electron microscopy (SEM), and Zeta potential analysis [22].

In summary, the aforementioned interactions may increase the utility of polyphenols by improving their stability and solubility, thereby further improving their bioavailability during gastrointestinal digestion. This value may ultimately translate into increased biological activity of the resulting components. Furthermore, these interactions influence the physicochemical properties of polysaccharides and the conformation and conjugation of proteins, impacting their practical applications in food, cosmetics, and medicine.

### 2.2. Impact on Health-Promoting Properties

The latest reports on gel agents and polyphenolic compounds focus on creating stable gel structures that protect bioactive compounds in order to fully utilize their health-promoting properties [23]. Therefore, it is crucial to understand the impact of these interactions on functional properties, which may have significant implications for the fields of food science, human nutrition, and medicine. Suspension of polyphenols in gelling substances may contribute to improving their stability by protecting them against degradation and enable modulation of their release in the gastrointestinal tract, which, depending on the properties of the system and digestive conditions, may affect the bioavailability and effectiveness of the health-promoting effects of these compounds [10]. This increases their bioavailability and the effectiveness of their health-promoting effects. Furthermore, interactions between polyphenols and the gelling matrix can positively influence the activity of polyphenols, enhancing their therapeutic effect. This is particularly important in the context of designing functional foods and dietary supplements [24]. The most frequently reported therapeutic effects include enhanced antioxidant, anti-inflammatory, anticancer, antimicrobial, antidiabetic and anti-circulatory system activity.

#### 2.2.1. Antioxidant Activity

Antioxidant properties are the most widely studied and recognized effects of polyphenolic compounds. These properties stem primarily from the large number of hydroxyl groups in their structure, which have been shown to scavenge free radicals and chelate metal ions [25]. By improving the stability and solubility of polyphenols as a result of their binding in the gelling matrix, their ability to transfer protons to free radicals is also increased, which leads to enhanced antioxidant activity [26]. For example, β-lactoglobulin (β-Lg) and gum arabic (GA) nanoparticles used to encapsulate epigallocatechin gallate (EGCG) have demonstrated a synergistic effect enhancing antioxidant activity. This was considered to be due to the increased stability of EGCG via hydrogen bonds with β-Lg and GA during simulated in vitro digestion [27]. Furthermore, interactions of polyphenols with gelling agents improve the water solubility of hydrophobic polyphenolic compounds, thereby increasing their radical scavenging activity. For example, the antioxidant activity of curcumin was nearly doubled when suspended in an alginate gel [28]. Additionally, suspending polyphenols in gelling agents is a good way to protect their antioxidant activity from the effects of high temperature and storage time. Tan et al. [29] demonstrated that encapsulation of black rice anthocyanins in pectin and their storage for 80 h at 50 °C reduced the loss of antioxidant activity in the chemical model by over 18.0%. Covering the capsules with a second layer of carboxymethylcellulose reduced the loss by another 5%. Packaging films, which act as a gel matrix for suspended polyphenols, also enable increased antioxidant activity and extended the shelf life of food through the controlled release of bioactive compounds [30]. Active coatings for fish fillets made of gelatin with incorporated conjugates of myricetin, kaempferol, and quercetin resulted in increased antioxidant activity, which contributed to the extension of the product’s shelf life [31]. This effect was stronger than when gelatin or polyphenols alone were used as a coating. The antioxidant activity of polyphenol–gelling agent components was also confirmed in vivo. In Wistar rats, administration of chitosan nanoparticles with a black carrot anthocyanin core resulted in an increase in serum catalase and superoxide dismutase activity [32]. Increased activity of antioxidant enzymes indicates an enhanced defense response against oxidative stress and the protective effect of the encapsulated compound [33].

#### 2.2.2. Anticancer Activity

Many reports have highlighted the importance of polyphenols in regulating cancer cell proliferation, which stems from their ability to modulate the cell cycle, induce cell death, and inhibit invasion and metastasis. Furthermore, as components of gelling agents, polyphenols represent a promising strategy for increasing therapeutic efficacy in cancer treatment [34]. The incorporation of polyphenols into the gel matrix allows many of their disadvantages to be overcome, which in turn leads to increased cellular uptake and more effective counteraction of drug resistance mechanisms [35].

A common way to target therapeutic systems to cancer cells is to use pH-sensitive gel matrices. Low pH (5.6) is a key characteristic of the tumor microenvironment [36]. Hence, Dutta et al. developed CuO-ZnO nanoparticles with bound *Trichosanthes dioica* fruit extract, coated with a pH-sensitive gelatin coating [37]. The drug release experiment demonstrated a pH-dependent release profile, with concentrated (99%) release at pH 5.6 and approximately 25% at pH 7.4. The formed nanocapsules also demonstrated a synergistic effect of the components used, enhancing cytotoxicity against cervical cancer cells (HeLa cell line). Protonation at low pH is also characteristic of chitosan polysaccharide [38]. For this reason, it was used to develop EGCG nanoparticle formulations targeting prostate cancer (line 22Rv1), inhibiting tumor growth and PSA (prostate-specific antigen) levels more effectively than free EGCG [39]. However, many types of cancer require multiple therapeutic approaches to maximize treatment outcomes. In this context, a gelatin hydrogel with chitosan nanoparticles loaded with curcumin was developed, which was sensitive to both pH and temperature. Under optimal conditions such as pH 5.0 and NIR radiation, curcumin release was 1.4 times higher than at pH 7.4 [40]. In addition to designing systems sensitive to endo- and exogenous factors, gelling agents can enhance the cytotoxic activity of polyphenols by modifying their hydrophobicity, improving delivery efficiency and cellular uptake [41]. One such polyphenol is quercetin, which has been proven to induce apoptosis of cancer cells by increasing the expression of pro-apoptotic proteins (Bad, Bax) and reducing the expression of anti-apoptotic proteins (Bcl-2, Bcl-xL) [42]. However, due to its low solubility, this compound is not a reliable choice for anticancer therapy. Hence, a recent study reported that gelatin nanofibers loaded with quercetin demonstrated a higher cytotoxic effect against liver cancer cells (HCT-116 cell line) than the free compound. The nanocomposite induced apoptosis and cell cycle arrest in the G0/G1 phase by increasing the mRNA expression of p53, p21, Bax, cytochrome C, and caspase-3 [43]. To improve the targeted delivery of anticancer drugs and reduce the side effects of therapy, research has been undertaken to develop their components with polyphenols, suspended together in gel materials. In this regard, 5-fluorouracil and methotrexate, combined with quercetin and encapsulated in chitosan, demonstrated synergistic effects by inducing intracellular production of reactive oxygen species (ROS), resulting in cancer cell apoptosis [44,45].

#### 2.2.3. Antimicrobial Activity

Polyphenolic compounds play an important role in maintaining food safety through their antimicrobial activity. This activity is primarily due to the presence of hydroxyl groups in their structure and electron delocalization, which interact with microbial organelles [46]. These interactions can affect metabolic pathways and lead to damage to cell membranes, proteins and nucleic acids of microorganisms [47].

Numerous studies suggest that conjugates of polyphenols with gelling agents may enhance their antimicrobial properties [48]. This may occur by protecting polyphenols from abiotic and biotic stressors such as temperature, light, pH changes, mechanical action, fermentation, and digestion. This effect was achieved through the use of gel carriers. Polysaccharides such as chitosan and pectin, and proteins such as gelatin, are most commonly used in this context [49]. Due to their good solubility, non-toxicity and biodegradability, these carriers are perfect for creating active food packaging [48]. For the active coating on apples made of quercetin with carboxymethyl chitosan, a 2-fold increase in the inhibition of mold and yeast growth was demonstrated [50]. An increase in polyphenol activity in chitosan or starch films has also been demonstrated in other studies [51,52]. Moreover, higher susceptibility to common foodborne pathogens such as *Escherichia coli* and *Staphylococcus aureus* was demonstrated after the use of hydrogels composed of gelatin with *Bletilla striata* polysaccharides and tea polyphenols, as well as pectin, whey protein concentrate and chlorogenic acid [53,54]. Also, edible coatings on apples and potatoes made from pectin and polyphenolic extract from apricot fruit (*Mimusopsis comersonii*) were able to reduce the level of aerobic mesophiles to 2 log CFU/g and also to reduce enzymatic browning [55]. In addition to their use in microbiological protection of food, polyphenols are also widely used to treat diseases associated with bacterial overgrowth, which is often the result of antibiotic resistance. One such condition is acne [56]. In one report, nanoliposomes with polyphenolic extract from algae (*Sargassum tenerrimum*) suspended in a film made of chitosan and sodium alginate were developed [57]. The films demonstrated higher MIC and MBC activity against acne-causing bacteria than the extract alone, resulting from improved stability and sustained release of polyphenolic compounds. Furthermore, coated polyphenols may bypass antibiotic resistance through various mechanisms, including increased production of intracellular ROS [58].

#### 2.2.4. Antidiabetic Activity

Recent studies indicate that polyphenols have antidiabetic effects through insulin-independent and insulin-dependent mechanisms, including protection of pancreatic beta cells, stimulation of insulin secretion, inhibition of glucose absorption, and modification of the inflammatory response [59]. Gel matrices can be used as materials to reduce glucose levels due to their ability to form a gel and bind sugars in the digestive tract [60]. In addition, these materials can regulate glycemia by protecting pancreatic beta cells, promoting insulin secretion, modulating intestinal microflora and resulting metabolites; they can also alleviate diabetic complications such as tetinopathy, nephropathy, and neuropathy [61,62].

Naringenin is a polyphenol with proven antidiabetic effects by improving insulin sensitivity, lowering blood glucose levels, and inhibiting monocyte chemotactic protein. However, due to its low water solubility and poor bioavailability, its therapeutic use is significantly limited [63]. Hence, nanoparticles with a naringenin core and covered with two layers composed first of chitosan and then of sodium alginate were developed [64]. Following oral administration to diabetic rats, a significant decrease in blood glucose levels was demonstrated, without toxic effects. This effect was explained by the stimulating effect of naringenin on beta cell islet regeneration, as well as the slow and long-term release of polyphenol from nanoparticles. Phenolic acids have also been shown to have high antidiabetic potential [65]. Hence, in one study, chitosan nanoparticles with ferulic acid were developed [66]. In an oral glucose tolerance test, Wistar rats demonstrated significantly lower blood glucose levels compared to the group treated with ferulic acid alone or the antidiabetic drug glibenclamide. Additionally, animals treated with nanoparticles experienced a 2.1% weight loss. Ferulic acid, as well as chitosan alone, may reduce diabetic hyperglycemia by increasing glucose uptake from skeletal muscle and inhibiting the activity of carbohydrate-hydrolyzing enzymes [60]. One of the undoubted advantages of gel materials is their high biocompatibility, which is why they are commonly tested in studies targeting diabetes complications. For example, alginate–chitosan nanoparticles have been shown to have anti-inflammatory properties, reducing complications associated with diabetic wounds [67]. In the treatment of wounds in patients with diabetes, dressings based on gel materials in the form of foil, hydrogel, nanofiber matrices or powders are widely used [68].

#### 2.2.5. Anti-Inflammatory Activity

The anti-inflammatory properties of polyphenols involve modulating molecular pathways and mediators responsible for inflammation. They can influence various levels of the inflammatory response, from cell signaling to gene expression to enzyme activity [25]. As a result, the potential of polyphenols in gel matrices to reduce inflammation and regulate the immune system response has become the subject of numerous studies [69,70].

Modifying gel materials to carry polyphenols can help enhance their functionality. For example, rutin was encapsulated in a chitosan hydrogel modified with 4-carboxyphenylboronic acid, which provided temperature-dependent adhesion and painless dressing removal [71]. In addition to its high biocompatibility and potent antimicrobial properties, this hydrogel demonstrated anti-inflammatory activity through the induction of anti-inflammatory markers (Arg1 and CD206) and inhibition of pro-inflammatory cytokine expression (TNF-α, IL-6), ultimately accelerating wound healing. The anti-inflammatory properties of chitosan have also been confirmed in in vivo studies. Raman et al. [72] used male Wistar rats with gastric ulcers to demonstrate the anti-inflammatory effect of chitosan nanoparticles with anthocyanin extract from black carrot. Oral administration of the nanoparticles alleviated inflammation by inhibiting the production of proinflammatory cytokines (interferon gamma, IFN-γ) and inducing the expression of anti-inflammatory cytokines (interleukin 4, IL-4). A significantly weaker effect was observed in the group tested with anthocyanins and empty chitosan nanoparticles alone. Another polysaccharide commonly used to organize bioactive polyphenol delivery systems is starch. Thanks to hydrogen bonds and hydrophobic interactions, it forms a stable composite with polyphenols [73]. Lutein-loaded lotus flower starch nanoparticles were developed [74]. The resulting nanoparticles enabled controlled release of lutein, demonstrating anti-inflammatory activity. The nanoparticles reduced ROS production, NF-κB pathway activation, and the secretion of interleukins (IL-1β, IL-6) and TNF-α. Notably, these effects were stronger than those observed with uncoated lutein and better in the prophylactic group than in the treated group.

#### 2.2.6. Impact on the Cardiovascular System

One factor that reduces the risk of cardiovascular disease is a diet rich in polyphenolic compounds. The most important cardioprotective mechanisms of polyphenols include reducing the level of asymmetric dimethylarginine (ADMA), regulating the antioxidant response factor (Nrf2-ARE), and inhibiting the activation of nuclear factor kappa B (NF-κB), which is sensitive to redox reactions and regulates the inflammatory response [75].

Protecting cardiomyocytes from cellular stress may be possible through the development of scaffolds composed of chitosan components combined with polyphenolic compounds. Chitosan may create a suitable environment for cardiomyocyte regeneration while reducing inflammation [76,77]. In this context, treatment of lipopolysaccharide-induced human endothelial cells with chitosan nanoparticles with a polyphenol-rich extract (standardized to gallic acid) led to a cardioprotective effect by reducing the levels of interleukin-6 (IL-6), TNF-α, and cyclooxygenase-2 (COX-2)-dependent prostaglandin E2 [78]. Chitosan was also used together with sodium alginate in another study to bind the polyphenolic fraction of *Olea ferruginea* leaves [79]. The resulting nanogel demonstrated efficacy in a rat model for the treatment of cardiovascular disease, primarily through antioxidant mechanisms through mitigating cardiac remodeling induced by excessive ROS concentrations. However, attempts to demonstrate the cardioprotective activity of polyphenols in carriers in humans have been unsuccessful due to low chemical stability and poor gastrointestinal bioavailability [23,80].

Analysis of the above reports indicates that the use of gelling agents to bind polyphenols can lead to increased biological activity, which is becoming an interesting area for development in food design and processing. However, future research should pay particular attention to and investigate other exogenous and endogenous factors influencing the relationship between polyphenol structures and gelling agents, which may modulate biological activity. Examples of reports examining other health-promoting properties of complexes of polyphenolic compounds with gelling agents are included in Table 1.

### 2.3. Bioavailability and Bioactivity of Polyphenolic Compounds

The bioavailability of polyphenols is the degree to which polyphenols contained in food are released from the food matrix, absorbed in the gastrointestinal tract, and available in the body to exert their biological effects. In turn, bioactivity is the ability of a compound (e.g., polyphenols) to cause specific biological effects in the body by interacting with cells, enzymes or metabolic pathways [94]. The value of these parameters is determined by many factors. It may depend on the given class or subclass of phenolic compounds, their physicochemical properties, the food matrix in which they are found, and the degree of technological processing (thermal or mechanical treatment) [95]. However, mainly due to their limited solubility and rapid metabolism, these compounds are commonly classified as substances with low bioavailability [96]. One potential solution to this problem is to suspend them in gelling matrices [15]. The use of such an approach may enable the protection of these compounds from unfavorable conditions in the gastrointestinal tract (e.g., low pH, action of digestive enzymes) and also promote their controlled release, improved solubility, and selective interaction with intestinal microbes [8]. Moreover, polyphenol–polysaccharidose and/or –protein complexes may constitute a favorable microenvironment, stabilizing the chemical structures of polyphenols, which directly translates into improved bioavailability, increased biological activity, and ultimately enhanced therapeutic effectiveness of these compounds [97].

#### 2.3.1. Protection Against Degradation in the Gastrointestinal Tract

So far, micro- and nanoencapsulation processes have been used to protect polyphenolic compounds from degradation in the gastrointestinal tract, including spray drying, freeze-drying, ionic gelation, coacervation, emulsion-based encapsulation, fluidized bed coating, and liposomal entrapment [17,98]. The function of these components after oral administration, depending on their final purpose, is to protect polyphenolic compounds against destabilizing factors, such as low pH of gastric fluid, alkaline pH of intestinal contents, action of digestive enzymes, and/or action of intestinal microflora [99]. Polyphenol protection occurs primarily through the physical barrier created by the gelling material. This improved stability and bioavailability of bioactive compounds allows for the preservation of polyphenol functionality compared to their untreated counterparts [100,101].

Gastric digestion may affect the bioavailability of polyphenolic compounds due to the action of pepsin and acid hydrolysis [102]. For some classes of compounds, an increase in concentration after digestion in the gastric phase by up to 45% is noted, which is the result of degradation into smaller units, mainly oligomeric flavonoids [103,104]. For others, such as phenolic acids, a decrease of 23–55% is observed, which is significantly dependent on the chemical structure, mainly the number of hydroxyl substituents of polyphenols [105,106]. However, it is generally accepted that it is more beneficial for polyphenolic compounds to reach the rest of the gastrointestinal tract in an unchanged state. One such method has been found to be suspension in microcapsules composed of sodium alginate and inulin carriers obtained by spray drying [107]. The stability of phenolic acids from propolis extract increased the stability of these compounds in the gastric phase by 15% compared to the free extract. Sodium alginate shrinks in a low-pH fluid, ensuring the stability of the microcapsules, but swells strongly at neutral or alkaline pH (intestinal fluid) [9]. Moreover, for nanocarriers composed of chitosan and pectin to protect anthocyanins from the influence of hydrochloric acid, a significantly lower release in the stomach (5%) than in the intestinal lumen (40%) has been proven [108]. Due to the fact that chitosan can dissolve in the stomach environment, the addition of pectin generated additional electrostatic interactions and hydrogen bonds, eliminating this disadvantage [17,109]. In another study, Xu et al. [110] developed a nanogel using calcium lactate-crosslinked alginate loaded with resveratrol to protect it from release in the stomach. The authors demonstrated that the technology used increased the stability of resveratrol in an acidic environment by more than 25% compared to the unencapsulated compound. Protection of polyphenolic compounds from degradation by gastric acid was not demonstrated compared to starch gels. According to a study by Yang et al. [111], a complex of gallic acid and resveratrol with rice starch gel released 60–80% of polyphenols in simulated gastric fluid. This was also confirmed in another study [112]. However, one study found that the kinetics of polyphenolic compounds’ release from the starch gel matrix is more dependent on the structure of polyphenols than on the pH value [113]. Moreover, it was found that noncovalent hydrogen bonds formed between polyphenols and starch influenced the textural properties of the gel, leading to the formation of a loose structure [114]. The most importance is attributed to the ability of polyphenols to increase the stretching of the polar covalent bond (O-H) of starch, which is crucial in the formation of hydrogen bonds [115].

The small intestine is the primary site of absorption of polyphenolic compounds. However, their chemical stability is also lowest there due to their high sensitivity to alkaline conditions, which favor their degradation [116]. Anthocyanins are the most sensitive to pH increases and undergo rapid degradation to form colorless chalcones [117]. Hence, in one of the articles, microcapsules were developed using sodium alginate combined with psyllium mucus to encapsulate polyphenolic extract from plum (*Carissa macrocarpa*) [118]. The results showed an anthocyanin bioavailability of 85.4% after the intestinal phase, significantly exceeding the value for microcapsules with alginate alone (66.4%). In another study, nanocapsules of chokeberry (*Aronia melanocarpa*) anthocyanins were developed in a chitosan gel, which led to a significant slowing of degradation and maintenance of antioxidant activity after simulated digestion in the small intestine [119]. Moreover, suspension of chokeberry extract in a mixture of gum arabic and maltodexrin also led to an increase in anthocyanin retention to a level of 75% compared to 58% for the pure extract [120].

Another important reason for the low bioavailability of polyphenols in the small intestine is oxidative degradation. The sensitivity of polyphenols to autoxidation is particularly high in intestinal conditions due to the presence of oxygen and transition metals [116]. One of the phenolic compounds highly sensitive to oxidation in a weakly alkaline environment is 2-aminobenzamide 2c (AV-2c), which is found in oats. As a result of its nanoencapsulation with a caseinate solution, its intestinal bioavailability more than doubled, and the cellular uptake rate in the Caco-2 model increased by 0.6% [121].

Moreover, it has been reported that an important factor influencing the low bioavailability of polyphenols in the lumen of the small intestine is the activity of hydrolytic enzymes, in particular intestinal esterases and glucosidases [116]. These enzymes can break down glycosidic bonds in polyphenols and lead to the formation of aglycones, which are less stable and more easily degraded [122]. In this context, microencapsulation creates a physical barrier that delays access of intestinal enzymes to polyphenols, which leads to an increased chance of absorption in their biologically active form. The use of gelling agents to prevent enzymatic degradation has been demonstrated for EGCG in chitosan and gelatin matrices, among others [123]. Also, embedding isoquercetin in a gelatin complex with okra polysaccharides allowed for the increased protection of isoquercetin against the acidic environment of the stomach and digestive enzymes [124].

Moreover, the low bioavailability of some classes of polyphenols is influenced by their low solubility, which limits their availability for absorption [125]. This mainly concerns curcumin, which, due to its crystalline and hydrophobic structure, is poorly soluble both in water and bile salts [126]. Studies have shown that suspending curcumin in a caseinate shell increased its solubility by more than 2500 times [127]. This increase in solubility was also confirmed for the gum arabic matrix [128]. The improved solubility of curcumin can be attributed to its amorphous structure within the encapsulating materials [129]. Moreover, suspending curcumin in nanocapsules led to an increase in its surface area, which facilitated its dissolution by bile salt micelles and thus increased its bioavailability [128].

In short, the problem of the low bioavailability of polyphenols can be overcome by suspending them in gel matrices. These effects result, on the one hand, from the improved solubility and protection provided by the carrier materials, ensuring their intact delivery to the absorption sites. On the other hand, cellular adhesion and nanoparticle size enhance absorption through intestinal membranes, improving bioavailability.

#### 2.3.2. Controlled Release

One promising solution for increasing the bioavailability of polyphenols is their controlled release from gel matrices. The goal is to maintain the active ingredient’s stability as much as possible during its passage through the gastrointestinal tract, with maximum release at the site where it is to be absorbed or fulfill its other function [116,130]. The most common technique used for controlled release of active substances is encapsulation, where the protection, transport, and controlled release of polyphenolic compounds takes place through reactions to various physiological factors, such as enzymes, pH and temperature [131]. The physicochemical properties of the carrier materials used have a key impact on the release kinetics of polyphenols, including their porous structure, ability to form gel networks, and sensitivity to environmental factors such as pH, the presence of digestive enzymes, and ionic strength. Numerous studies are currently being conducted on the use of gel matrices for targeted delivery of polyphenols to the large intestine [132].

The delivery of bioactive compounds to the large intestine causes many difficulties due to the need to overcome various barriers within the digestive tract [133]. These include changes in pH, type, and level of enzyme activity; transit time; volume of digestive fluids; mucus structure; and variability in the gut microbiota [134]. Nevertheless, many studies have proven measurable health effects after maintaining a high concentration of polyphenols in the colonic lumen [94,133]. In this context, we mainly talk about proanthocyanidins and anthocyanins, which, when they reach the large intestine intact, have a beneficial effect on colonocytes, protecting against inflammatory diseases and colon cancer [135]. One study also found an increase in the antioxidant activity of polyphenols as a result of metabolism by gut microflora [136] as well as a beneficial effect on the gut microflora itself. For these reasons, many authors have sought to develop oral formulations that modulate the bioavailability of polyphenols by protecting them from chemical degradation in the upper gastrointestinal tract, with peak release in the colon. In one study, a nanogel based on sodium alginate and selenized konjac glucomannan was developed for targeted and pH-dependent delivery of blueberry (*Vaccinium corymbosum*) anthocyanins to the large intestine [137]. The nanogel disintegrated and released anthocyanins only upon contact with intestinal fluids, demonstrating a mucoadhesive effect. It has also been shown that polyphenols not absorbed in the stomach and small intestine can reach the colon, where they interact with the gut microbiota, modulating its composition and metabolic activity, including participating in the regulation of microbial metabolic pathways and indirectly promoting the production of short-chain fatty acids (SCFAs). The produced SCFAs serve as energy for intestinal cells, while also positively influencing intestinal homeostasis. Therefore, in one study, sodium alginate and chitosan nanoparticles were developed with a polyphenolic extract from Siberian apple (*Malus baccata*) [138]. Studies in mouse feces revealed more than twice the concentrations of acetic acid, n-butyric acid, and propionic acid for nanoencapsulated polyphenols, which resulted from the polyphenols being delivered intact to the colon. Carboxymethyl chitosan with gallic acid also demonstrated a significant effect on the colonic microbiota, including an increase in the levels of *Enterococcus* and *Enterobacter* strains and a decrease in the levels of *Escherichia* and *Shigella* [139].

In recent years, active targeting of micro- and nanoparticles has become one of the treatment methods for colon cancer. This involves decorating their surfaces with various ligands, which allow the nanoparticles to accumulate in tumors through interactions with receptors [140]. A characteristic feature of cancer cells is the overexpression of various receptors, and in the case of colorectal cancer, the most well-known are folate receptor, transferrin receptor, and epidermal growth factor receptor (EGFR) [141]. Hence, one study designed nanoparticles of guar gum with curcumin, decorated with folic acid on the surface. The study showed that curcumin release in colonic fluid was 74%, while in gastric fluid, it was only 12% [142]. Generally speaking, guar gum is considered one of the most important matrices for the design of carriers targeting the colon due to its non-ionic and uncharged properties, which prevent its disintegration in the upper gastrointestinal tract but cause its degradation by the colonic microbiota [143].

Based on the above literature, it can be concluded that gelling agents constitute a valuable matrix for optimizing the efficiency of polyphenol delivery to the colon, taking into account factors such as digestive enzymes, pH, and gut microbiota. Their interactions highlight the potential of these systems for targeted prevention and/or treatment of chronic colonic diseases. Importantly, future human clinical trials confirming targeted delivery and beneficial effects on colon health should also be considered in this context.

### 2.4. Interactions of Polyphenols with Gelling Substances in Food Processing

Interactions between polyphenols and proteins and polyphenols and polysaccharides influence the structure and texture of food products, which translates into improved functionality and quality. These effects result primarily from the synergistic interaction of polyphenols with selected macromolecular components [21]. Food products based on polysaccharides and proteins enriched with polyphenolic compounds exhibit increased storage stability and improved quality properties, highlighting the importance of these interactions in designing foods with enhanced utility. These properties have applications in food preservation and the production of bioactive packaging, in the production of hydrogels and nanocomplexes, and in improving starch properties, such as modifying pasting, gelatinization, and retrogradation properties [144,145].

In products such as jellies, jams, and jellies, whose gel structure is based on gelatin or gelling polysaccharides, polyphenols contribute, among other things, to supporting gel formation and its structural integrity. These properties are utilized in products where the creation of a firm and cohesive cardboard is desired [146]. Polysaccharides with gelling properties (e.g., starch, agar and pectin) combined with polyphenolic compounds create a stable texture, and the gels are elastic or springy [147,148]. In foods enriched with phenolic compounds, the most frequently studied interactions are those with proteins and saccharides, because those interactions influence the sensory and technological properties of food products [149].

#### 2.4.1. Polyphenol–Protein Interactions

Protein gels are three-dimensional network structures that entrap a liquid. This structure is supported by intermolecular covalent and noncovalent protein bonds. The preference for noncovalent or covalent interactions depends on the conditions under which the interactions occur as well as the initial properties of the proteins or polyphenols. The challenge in studying such interactions lies in the structural diversity of proteins, as well as the diversity of polyphenolic compounds [149] There are different types of gels, but the most common ones found in food are those induced by heating, protein denaturation, and cross-linking. Proteins subjected to high temperatures form gels in three stages: (I) protein unfolding and dissociation, (II) aggregation of unfolded proteins, and (III) interaction of aggregates to form a space-filling gel network [150].

Noncovalent interactions between proteins and polyphenols are mainly stabilized by a combination of reversible hydrophilic and hydrophobic interactions (including hydrogen bonds, ionic or electrostatic interactions, and hydrophobic stacking interactions). The van der Waals forces with nonaromatic hydrophobic protein side chains of some amino acids (e.g., isoleucine, leucine) make a small contribution to the interactions [151]. The covalent bond in the protein–phenol system is formed primarily through the oxidation of a polyphenolic compound, followed by coupling reactions. The most well-known mechanism of the protein–phenol reaction is the Michael addition of nucleophilic groups from the protein side chains to electron-deficient or p-quinones resulting from the oxidation of phenolic compounds. The physicochemical conditions influencing protein–phenolic interactions are temperature, pH value, and ionic strength [151]. Another important factor is the chemical structure of phenols. A noncovalent interaction with proteins is stronger for polyphenolic compounds that contain multiple phenolic moieties. Polyphenolic compounds may affect the protein’s technological properties, e.g., gelation process, foam or emulsion stabilization [151].

##### Interactions of Polyphenols with Myofibrillar Proteins in the Form of Gels

Myofibrillar proteins (MPs) are primarily responsible for the textural properties of processed meat products. Myosin and actin in myofibrillar proteins contribute most to the development of desirable gel properties in processed meat products [146]. Thermal gelation of myosin creates a three-dimensional network structure that maintains water in a less mobile state. During network formation, fat and water retention increases, which affects the yield, texture, and consistency of the final product and also determines the gelling ability of MP proteins. During food processing, myofibrous proteins are susceptible to free radical attack, leading to changes in their physicochemical and functional properties, such as gelling ability. Therefore, oxidative stress-induced modification of MP proteins is currently widely studied and discussed in food and technology [152]. The concentration of polyphenols in the polyphenol–protein system plays a key role in the formation of gels from myofibrillar proteins. Too low a concentration contributes to the participation of a larger number of active groups in gel formation and increases protein aggregation but does not significantly regulate protein cross-linking and aggregation. High polyphenol concentrations, on the other hand, can cause excessive and disordered protein aggregation and cross-linking, resulting in protein precipitation and the destruction of their structure [153]. Rutin at low concentrations (10, 50, 100 μmol/g) can improve gel strength but reduce water retention capacity. Higher rutin concentrations (200 μmol/g) can have a negative effect on the gel in question. However, regardless of the rutin concentration used in the myofibrillar protein (MP) gel, the samples were characterized by a higher final elastic storage modulus and transformed into gels with dominant elasticity. This indicates that higher rutin concentrations can improve MP gel properties, which is related to rutin-induced protein conformational changes [154]. Jiang et al. [155] added three polyphenols—including gallic acid (GA), quercetin (QR), and rutin (RH)—to goose myofibrillar protein (GMP) to explore effects on the gel properties and structures of GMP. After adding polyphenols, the water holding capacity (WHC) and texture characteristics of GMP gel were significantly improved. In one study, based on the noncovalent interaction between polyphenols and proteins, all three polyphenols improved the gel properties of GMP, with QR demonstrating the most significant effect, which was associated with structural changes in GMP.

Cheng et al. [156] evaluated the effects of five dominant phenolic compounds (cyanidin 3-*O*-glucoside, cyanidin 3-*O*-rutinoside, caffeic acid, quercetin, and rutin) presented in mulberry polyphenol-enriched sausage on the configuration and functionality of MP. The studied phenolic compounds significantly affected the structure of the myofibrillar protein. All phenolic compounds were involved in the changes in meat product quality, but caffeic acid and rutin were the most critical ones. Modified myofibrillar protein increased antioxidative activity and decreased thermal stability. Rutin did not affect myofibrillar protein in thermal stability but improved emulsifying properties. Quercetin had little effect on the secondary structure of myofibrillar protein. Caffeic acid resulted in protein exhibiting the strongest solubility and antioxidant activity among all samples. Ni et al. [157] investigated the impact of tea polyphenols (epicatechin, epigallocatechin, and epigallocatechin gallate) on the structural changes, emulsifying properties, water-holding capacity, and gel characteristics of myofibrillar protein of freshwater fish species under oxidative conditions.

All three tea polyphenol compounds slowed down the oxidation process of myofibrillar proteins and decreased the production of oxidative products. Moreover, tea polyphenols optimized gel strength, improved water holding capacity, and altered rheological properties. Epigallocatechin gallate, due to its unique chemical structure with more phenolic rings, formed complexes with myofibrillar proteins that exhibited greater antioxidant activity. Tea polyphenols can be a natural antioxidant in modifying surimi products, offering theoretical and technical support for the development of high-quality aquatic products. Leicht et al. [158] conducted by preparing myofibrillar protein gels enriched with polyphenol-rich plant-based additives (blackcurrant juice, blackcurrant pomace, Melissa officinalis extract, and Centella asiatica extract). The addition of M. officinalis enriched the product in anthocyanin and significantly improved the color and antioxidant capacity of myofibrillar protein samples.

The inclusion of plant extracts in products such as myofibrillar protein gels is currently an increasingly common trend in research on food enrichment and their impact on product quality. The use of plant extracts rich in polyphenolic compounds, which exhibit antioxidant properties, contributes to increased nutritional value of meat, poultry, and other products, as well as microbiological stability and sensory properties.

##### Protein Gels’ Interactions with Polyphenols in Dairy Products

Incorporating polyphenolic compounds into dairy products is one method for improving their technological functionality and nutritional value. This is due to the fact that proteins readily interact with polyphenols, primarily through hydrogen bonds and hydrophobic interactions. These interactions are primarily pH-dependent and are observed at the isoelectric point. Polyphenolic compounds or polyphenolic-rich extracts can be ideal ingredients for adding to dairy gels, as they can improve nutritional value and rheological properties [159]. Harbourne et al. [160] studied the effect of simple phenols (gallic acid) and hydrolysable tannins (tannic acid) on the rheological properties, changes in water mobility, and syneresis of acid milk gels. The addition of simple phenols and hydrolysable tannins influenced the rheological properties and water mobility of acidified milk gels and formed a strong gel. The rheological properties and water mobility in milk systems with acidified gels can be additionally influenced by introducing other gel-like substances, such as modified starches [161] or pectin [162].

Some studies show that polyphenols can weaken gel network formation; for example, compounds contained in tea contributed to the formation of weak pea protein gels [163]. In the study by Jia et al. [164] the addition of chlorogenic acid to sunflower protein had a negative effect on gel strength, similar to the case of canola protein gels using sinapic acid or thomasidioic acid [165].

Noncovalent interactions between proteins and polyphenolic compounds appear to hinder gel network formation, but covalent interactions at lower levels tend to produce stiff gels. Covalent interactions in protein gels with polyphenols (polyphenol–milk protein complexes) become firmer and thermally stable and can produce the enhanced gelation properties of the complexes. The protein in whey, β-lactoglobulin, is the primary gelling agent that dominates the thermal behavior of the entire whey protein system. This lactoglobulin modified by green tea polyphenols exhibited an increased gelation property when the gelling temperature and gelling time were decreased [166]. Han et al. [167] produced milk gels with polyphenolic compounds (including catechin, epigallocatechin gallate, tannic acid, homovanillic acid, hesperetin, and flavone) along with natural crude compounds (such as grape extract, green tea extract, and dehydrated cranberry powder). Cheese curds containing polyphenolic compounds at a concentration of 0.5 mg/mL showed a decrease in curd moisture content, but the gel strength was not affected. The addition of polyphenolic compounds resulted in rough and granular structures of the cheese.

#### 2.4.2. Interactions of Polyphenols with Starch Gels

Starch is a carbohydrate consisting of two types of chains—amylose and amylopectin—whose characteristic phenomenon is retrogradation [168]. Starch heated in an aqueous environment undergoes gelatinization, meaning that starch granules in excess water undergo a transformation during which water diffuses into the granules, swelling them, followed by loss of starch granule crystallinity and leaching of amylose into the aqueous environment. After the gelatin cools, the amylose and amylopectin chains form a partially ordered gel structure [169,170]. Both amylose and amylopectin influence this process, contributing to what is known as short- and long-term starch retrogradation, respectively. The retrogradation of starch pastes and gels is both a desirable and undesirable process in food products. Due to changes in food consistency and the occurrence of syneresis, food texture undergoes unfavorable changes [171]. From a nutritional perspective, the so-called resistant starch (RS) is formed, which acts as a dietary fiber and prebiotic [172,173].

Wu et al. [174] studied the effect of tea polyphenolic compounds on rice starch retrogradation using X-ray diffraction and differential scanning calorimetry. According to the cited authors, these methods can demonstrate whether the addition of polyphenolic compounds can extend the shelf life of starch-based products. The highly reactive -OH groups contained in tea compounds interact with the OH groups of starch, forming hydrogen bonds (hydrogen bridges), which disrupt the alignment of starch chains. According to Wu et al. [174], the important aspect here is the reactivity of OH groups, not their number, as they believe [175]. Zhu et al. [176] added 25 polyphenolic compounds with different molecular structures to wheat starch. All phenolic acids caused a decrease in the hardness and adhesion of starch gels. However, chrysin, trans-chalcone, and trans-stilbene increased the hardness and adhesion. Moreover, most flavonoids did not affect the adhesion of starch gels [177]. As assessed by DSC and X-ray diffraction, the addition of various plant extracts and pure polyphenolic compounds has a strong influence on starch retrogradation to varying degrees [174,178,179]. A series of studies showed that the effect of tea polyphenols on starch retrogradation was dependent on the type of starch and the concentration of polyphenolic compounds. While most studies found that tea polyphenols inhibited the retrogradation of starches of various botanical origins [174,179], another study showed that black tea polyphenol extract reduced the retrogradation of rice and corn starch and had little effect on this property in potato starch [178]. The hydroxyl groups of polyphenols can interact with water and the hydroxyl groups of starch through hydrogen bonds, thus preventing the retrogradation of starch [180,181].

## 3. Methods

Reports required for this article were searched in PubMed, Google Scholar, and ScienceDirect. The search was conducted between July and October 2025. A MeSH database and keyword strategy were used to electronically search for articles. A combination of relevant keywords and phrases was used: “Gelling Agent,” “Gelling Substances,” “Food Gels,” “Gelation,” “Hydrogels,” “Gels,” “Microcapsules,” “Nanocapsules,” “Fibers,” “Polyphenolic Compounds,” “Health-Promoting Properties,” “Bioactivity,” “Bioavailability,” “Proteins,” “Polysaccharides,” “Texture,” “Rheology,” “Gel Stability,” and “Gelation Mechanism.” Inclusion criteria included original and review articles published in peer-reviewed journals; studies directly related to gels or their formulations and their applications in the aforementioned areas; and papers available in full text. Exclusion criteria included non-scientific publications; articles outside the scope of the topic; and conference proceedings without full text. The article selection process was conducted in three stages: identification, initial screening based on title and abstract, and analysis of full texts according to the adopted criteria. A total of 482 records were identified from database searches; after removing duplicates and items that did not meet the initial criteria, 232 articles were considered for full-text review, of which 183 were ultimately included in a detailed narrative analysis. The quality assessment of the included studies was based on the following criteria: methodological reliability, relevance to the review questions, and the relevance of their findings to the discussed applications of gels and functional materials. Articles with low methodological quality or insufficient description of methods were omitted from the synthesis of results to ensure the reliability and substantive value of the final conclusions.

## 4. Summary and Conclusions for Future Research

The novelty of this work is the comprehensive discussion of recent advances in enhancing the functional properties of polyphenolic compounds by binding them in gelling agents. Over the years, gelling agents have gained importance as effective matrices enabling the optimization of the functional properties of phytochemicals. Their use in the form of hydrogels, microcapsules, nanocapsules, films, membranes, or fibers can overcome the problems associated with polyphenolic compounds related to low bioavailability, solubility, autoxidation, and stability, contributing to the development of innovative and effective delivery systems for bioactive ingredients and advanced functional food formulations.

The suspension of polyphenolic compounds in gelling systems also creates a number of significant challenges that should be addressed in future research. One challenge is the production costs of these formulations, which require the use of specialized equipment and high energy consumption, which can ultimately generate higher production costs compared to traditional methods. From a sustainable development perspective, it is also crucial to develop eco-friendly, energy-efficient synthetic methods that incorporate the use of waste and renewable raw materials while maintaining economic viability. Another important factor is the need to develop standardized procedures for the synthesis and conjugation of polyphenols with gelling agents to obtain reproducible results and enable the development of scalable technologies. Finally, this development should include the confirmation of results in in vitro and in vivo models, emphasizing the need to develop more representative gastrointestinal models to confirm the effectiveness of improving the bioavailability and bioactivity of phytochemicals as well as conducting clinical trials. In this context, it is also crucial to deepen toxicological studies and assess the biocompatibility of the developed systems; it is also necessary to consider the impact of long-term use on human health. All of the above challenges constitute a necessary step towards the full commercialization and application (both food-based and medical) of polyphenolic compounds bound in gelling systems.

## Figures and Tables

**Table 1:** Example of reports examining other health-promoting properties of complexes of polyphenolic compounds with gelling agents.

No	Action	Gelling Agent	Polyphenolic Compounds	Form	Demonstrated Activity	References
1	Hepatoprotective	Chitosan	Silymarin	Nanoparticles	Enhancement of silimatin bioavailability; improvement in inhibition of aluminum (Al)-induced liver damage; reduction of ALT, AST, ALP and LDH.	[81]
2	Hepatoprotective	Gum arabic, maltodextrin-dextrose	Chokeberry extract (*Aronia melanocarpa*)	Microcapsules	Enhanced protection against liver damage caused by a high-fat diet; decrease in ALT, AST, TC TG, and LDL-C.	[82]
3	Hepatoprotective	Microcrystalline cellulose, wheat starch	Common knotweed extract (*Polygonum equisetiforme*)	Microcapsules	Improved inhibition of nickel (Ni)-induced damage; reduction of AST, ALT, ALP, and LDH.	[83]
4	Hepatoprotective	Starch, bovine serum albumin	Moringa oleifera leaf extract (*Moringa oleifera* Lam)	Nanoparticles	Enhancement of inhibition of liver damage caused by bisphenol A; reduction of ALT, AST, and ALP after treatment with bisphenol A; improvement of the distribution and density of liver collagen fibers.	[84]
5	Hepatoprotective	Pectin, polyacrylic acid	Indian horse chestnut extract (*Aesculus indica*)	Hydrogel	Increased effectiveness in reducing liver fibrosis; reduction of inflammatory infiltration after CCl_4_ treatment.	[85]
6	Neuroprotective	hyaluronic acid, collagen	Pomegranate polyphenol extract (*Punica granatum* L.)	Hydrogel	Enhancement of stimulation of neuronal growth and differentiation.	[86]
7	Neuroprotective	Carboxymethyl chitosan	Tannic acid	Hydrogel	Sustained release of tannic acid; enhancement of neuroplasticity after stroke.	[87]
8	Neuroprotective	Chitosan	Carnosic acid	Nanocapsules	Improving the protective effect of carnosic acid against toxic concentrations of H_2_O_2_.	[88]
9	Atopic dermatitis	Hyaluronic acid, chitosan	Resveratrol	Hydrogel with nanoparticles	Controlled release of resveratrol; reduction of IL-4, IL-6 and IL-33 secretion; inhibition of ROS production by keratinocytes.	[89]
10	Wound healing	Gum arabic, apple pectin	Naringenina	Hydrogel	Acceleration of wound healing through increased angiogenesis, reepithelialization and collagen deposition.	[90]
11	Wound healing	Sodium alginate	Ribwort plantain extract (*Plantago major* L.)	Microcapsules	Accelerated wound healing; reduction of IL1α and IL1β.	[91]
12	Dermatitis	Sodium alginate, chitosan	Olive waste extract (Olea europaea)	Microcapsules	Attenuation of the release of TNF-α, IL 6, IL1β and IL 12.	[92]
13	Anti-aging	Chitosan	Blueberry fruit extract (*Vaccinium myrtillus*)	Hydrogel	Improved inhibition of hyaluronidase and tyrosinase activity.	[93]

## Data Availability

No new data were created or analysed in this study. Data sharing is not applicable to this article.
